# Characterization of Feeding, Sport Management, and Routine Care of the Chilean Corralero Horse during Rodeo Season

**DOI:** 10.3390/ani9090697

**Published:** 2019-09-17

**Authors:** Joaquín Bull, Fernando Bas, Macarena Silva-Guzmán, Hope Helen Wentzel, Juan Pablo Keim, Mónica Gandarillas

**Affiliations:** 1 Departamento de Ciencias Animales, Facultad de Agronomía e Ingeniería Forestal, Pontificia Universidad Católica de Chile, Avda. Vicuña Mackenna 4860, Santiago 7820436, Chile; jabull@uc.cl (J.B.); fbas@uc.cl (F.B.); 2 Private statistical consultant, Guardia Vieja 441, Santiago 7510318, Chile; maca.silva.guzman@gmail.com; 3 Escuela de Graduados, Facultad de Ciencias Agrarias, Universidad Austral de Chile, Valdivia 5110566, Chile; hope.wentzel@alumnos.uach.cl; 4 Instituto de Producción Animal, Facultad de Ciencias Agrarias, Universidad Austral de Chile, Independencia 641, Valdivia 5110566, Chile; juan.keim@uach.cl

**Keywords:** Chilean corralero horse, rodeo, feeding practices

## Abstract

**Simple Summary:**

The Chilean corralero horse holds great cultural importance due to its use in Chilean rodeo, the national sport. However, information regarding this breed is sparse, especially husbandry, feeding, and training recommendations, which could present challenges for their proper care. A survey of horse farms in several regions from central to southern Chile was conducted in order to document current management of the Chilean corralero horses which participated in the 2014–2015 Chilean Rodeo Federation season. In the survey, horse owners and trainers were asked about horse gender and size, daily routine, exercise and competition regimen, and feeding practices. All horses in the study were kept in stalls for at least 12 h daily and spent the rest of the day either tied or loose in pens or paddocks. Horses were in moderate- to high-intensity exercise programs, with workouts six days/week and two rodeos per month. Feeding practices varied greatly among farms but most horses received forage (alfalfa or grass hay) and an energy feed (oats, corn, or concentrate), while protein and lipid supplements were less common. The goal of this characterization of current management of the Chilean corralero horse is to contribute to information available about this breed to improve husbandry practices.

**Abstract:**

The aim of this study was to characterize the routine care, training, feeding, and nutritional management of Chilean corralero horses that participated in the rodeos of the Chilean Rodeo Federation. Forty-nine horse farms between the Metropolitan (33°26′16″ south (S) 70°39′01″ west (W)) and Los Lagos Regions (41°28′18″ S 72°56′12″ W), were visited and a survey was conducted on the management and feeding of the Chilean horse. Of the horses which participated in at least one official rodeo in the 2014–2015 season, 275 horses were included in the study. The survey consisted of five questions about general data on the property and the respondent, four questions on the animal characteristics, five questions about where the horses were kept during the day, seven questions to characterize the amount of exercise done by the horse, and 18 questions about feeding practices; additionally, the amount of feed offered was weighed. All horses in this study were in training and kept in their stall for at least 12 h and remained tied or loose for the rest of the day. The intensity of daily exercise of the rodeo Chilean horse could be classified as moderate to heavy and consisted of being worked six days/week and participating in two rodeos/month. Ninety-eight percent of respondents had watering devices in the stables. The diet of the Chilean corralero horse during the training season is based on forages, mainly alfalfa hay, plus oats as an additional energy source. Protein supplements such as oil seed by-products are used less frequently. A wide variation was observed in the diets and quantities of feed offered, which suggests that the feeding management of these individuals is not formulated according to their requirements.

## 1. Introduction

Horses served humans for centuries as a source of food, as well as for military, agricultural labor, and sport purposes [1]. The International Federation for Equestrian Sports (FEI) recognizes eight disciplines for competition (dressage, jumping, vaulting, endurance, reining, combined driving, eventing, and para-equestrian). Nevertheless, there are several other widely known equestrian sport competitions worldwide, such as polo and racing.

In Chile, “rodeo” was declared the national sport in 1962 (Decree 269 of the National Council of Sports), when it became part of the Olympic Committee of Chile [2]. This sport is one of the culturally rich activities that take place in the Chilean countryside. It symbolizes traditional cattle-working and is part of the traditional folklife with its own customs [2,3]. After football (soccer), rodeo is the second most popular sport in Chile due to its massive following and because it became an important cultural symbol throughout national history.

The breeding aim for the Chilean corralero horse is to produce a horse that is suitable and functional for saddle and stock work, as well as practicing rodeo [4].

The rodeo is performed in an oval-shaped area and consists of a pair of riders (each rider is called a “huaso”) and their horses (“collera”) running half laps around the arena while working a steer and attempting to pin it against a large 12-m cushion (“quincha”). Horses run a total of approximately 400 m at 6.95 m/s in each round of the competition [5]. The rodeo is highly regulated by the Chilean Rodeo Federation, and there are 311 officially registered arenas throughout the national territory [6], with 350 competitions per season, which begins in August each year and ends with the national championship in April of the following year.

Despite the national importance of rodeo, there is little information available and easily accessible about the Chilean corralero horse, specifically related to equine numbers, competing animals, feeding type, and routine management. Moreover, unlike other equestrian sports like show-jumping [7], racing [8], and three-day eventing [9], there is no known characterization of feeding management and schedule or dietary ingredients. Since competition horses may develop nutritional and digestive problems such as gastric ulcers, decreased appetite, and weight loss [10], it is important to establish feeding practices of sport horses undergoing hard work. Considering that the rodeo sport season lasts eight months every year, with some horses competing every weekend or every other weekend, a properly designed training routine and sound feeding management is crucial. Moreover, since the Chilean corralero horse is considered a small horse (height at the withers 99.86–136.4 cm) and *huasos* weigh an average of 80.78 + 10.02 kg, some of these horses may be overburdened [5], thus requiring additional energy.

The lack of information on Chilean corralero horses also makes it difficult for breeders, trainers, riders, veterinarians, and owners to develop management and feeding standards. Therefore, this work seeks to contribute with relevant information on diet composition and feed ingredients utilized daily in the diet of Chilean corralero horses actively participating in the national sport. Hence, the objective of this study was to characterize the feeding and nutritional management, routine care, and training of Chilean corralero horses that participate in the rodeos of the Chilean Rodeo Federation.

## 2. Materials and Methods

### 2.1. Animals

The target population for this study was healthy Chilean corralero horses that participated in the 2014–2015 season of the Chilean national rodeo competition and that were in training at the time of the visit. The data to identify individuals that participated during that season were provided by the Chilean Rodeo Federation.

The horses’ owners were contacted by telephone or e-mail to request their participation in the study, and an interview was scheduled if they were willing to participate. As a result, a personal visit was carried out between October and December of 2015 to 49 farms (275 horses) located between the Metropolitan and the Los Lagos regions (33°26′16″ south (S), 70°39′1″ west (W) to 41°28′18″ S, 72°56′12″ W). Farms were located in the following regions: Metropolitan (*n* = 5), Valparaiso (*n* = 3), O’Higgins (*n* = 6) Maule (*n* = 5), Bío-Bío (*n* = 10), Araucanía (*n* = 8), Los Ríos (*n* = 5), and Los Lagos (*n* = 7).

### 2.2. Survey Design

The interview consisted of a questionnaire with five questions regarding general farm and respondent information (farm name, location, farm administrator, position, date of the visit), three questions to characterize the animal (registration number, sex, date of birth), five questions about housing conditions and activity (hours spent within the stall, outdoor pen, paddock, and tied), and four questions characterizing the type and amount of daily exercise of the horse (minutes spent walking, trotting, cantering, and in the “bumping activity”, on a weekly and daily basis). “Bumping” (topeo) is when the horse performs lateral canter half-passes while maintaining contact at a 45° angle between the horse’s chest and the steer’s body, using a lateral movement to push slightly on the steer and maintain him at a 45° angle to the horse’s body.

The questionnaire also included 18 questions about nutritional management with regard to feeding (feed ingredient type, feeding frequency, and schedule), water availability and source (automatic drinker, water bucket, etc.), access to pasture, hay type, grains, protein supplements, other commercial concentrate or byproduct, minerals, oils, if feedstuffs were moisturized, nutritional advisor, and other information (de-wormer type and frequency, incidence of colic and laminitis per year).

The daily amount of each ingredient offered was weighed with a digital scale. The girth and scapula–ischial length (SIL) were measured using a measuring tape to estimate live body weight, using the formula of Carroll and Huntington [11], where

weight (kg) = (girth (cm^2^) × SIL (cm))/11,877. (1)

The girth corresponds to the thoracic perimeter (cm) and was measured by wrapping a 3-m flexible measuring tape around the girth, just behind the withers, of each horse. The scapula–ischial length (SIL) corresponds to the length from scapula (point of the shoulder) to ischial tuberosity (point of the buttock) in centimeters [12].

The unique registration number and date of birth of the horses were obtained from the owners or the animal’s registration certificate.

### 2.3. Statistical Analyses

Data obtained from the surveys were consolidated in an Excel spreadsheet and reported as a descriptive analysis (average ± standard deviation, frequency, average, minimum, maximum). One-way ANOVA between horses was conducted to determine significant differences among treatments, and a Tukey honestly significant difference (HSD) test was performed whenever there were significant differences. Significance was declared with a *p*-value lower than 0.05. Pearson correlations were performed between feedstuffs (hay, corn, oats) utilized and incidence of colic and laminitis. The Pearson correlation was performed among different exercises types (walk, trot, canter). Data were processed using Statistica V 7.0.

## 3. Results

Measurements were collected from 275 horses located on 49 farms, and general characterizations about gender, age, and physical characteristics are presented in Table 1. The evaluated farms had geldings, mares, and stallions, which respectively accounted for 30.6%, 32.7%, and 36.7% of the entire sampled population. The average age per horse was 9.9 ± 2.6 years old. Geldings in this survey were older than mares and stallions (*p* < 0.05).

The estimated weight of mares was greater than males (geldings and stallions) (*p* < 0.05), but there was no significant difference between gelding and stallions. When all horses were considered, the average estimated weight was 383.7 ± 26.8 kg. The averages of girth and SIL per horse were 166.7 ± 4.3 cm and 151.8 ± 5.3 cm, respectively, with mares having a larger girth compared to stallions and geldings (*p* < 0.05) and a larger SIL compared to geldings (*p* < 0.05).

Routine care did not follow a common pattern among farms; however, all horses were kept in individual stalls during the night and for part of the day. Eighty-six horses (31.3%) spent the entire day in stalls. Of the rest, 163 horses spend part of the day tied, 24 in pens, and two in paddocks (Table 2).

The sport and exercise management routine of the Chilean corralero horses is presented in Table 3.

Each farm studied had a unique routine for each of their horses. On average, horses trotted for 8.6 ± 6.6 min/day and cantered for 22.3 ± 6.1 min/day. All farms utilized bumping as an exercise, usually for durations of between 10 and 20 min and two times/week. Lunging was not a common practice among trainers; only 6.5% of horse were lunged for more than 15 min. A Pearson correlation (*r* = −0.44) was detected between trot and canter exercises, indicating that the time spent on one of the two exercises is inversely correlated to the other. There was no correlation detected between exercise routine and number of rodeos annually. The average number of rodeos during the season was 8.6 ± 4.7 per horse; 20 (41.0%), 16 (33.0%), and five (10.0%) farms participated in two, three, and four rodeos per month, respectively. Eight farms participated in one or fewer than one rodeo per month during the 2014–2015 season.

Feeding and nutritional management of the Chilean corralero horse during the rodeo season is summarized in Table 4. Results showed that 81.1% of the horses were fed twice daily (morning and noon/night), and 18.9% received three meals (morning, noon and afternoon/evening). The horses that had two rations per day ate during the morning and night (212 horses from 37 farms) or noon and night (11 horses from two farms). None of the horses surveyed had access to pasture.

A total of 265 horses were fed hay. Each studied farm used more than one hay type to feed their horses. The average amount of hay offered per horse was 9.0 ± 1.9 kg/day as fed. Alfalfa hay was the most commonly used forage, followed by a pasture hay and alfalfa cubes/pellets. Twenty-seven percent of horses received some type of cereal grains. Oats were the most commonly used grain, followed by corn which was offered fine, ground, or rolled.

Wheat milling by-products were also part of the feed used, where 61.2% of the farms used middlings or bran. The delivered amount of wheat bran ranged from 0.5 kg/day to 5.3 kg/day, whereas wheat middlings were offered at a range of 0.7 to 6.0 kg/day.

A total of 38 farms (77.6% of farms surveyed) did not use oilseed by-products (soybean and canola meal) to feed any of their horses. Eleven farms utilized oilseed by-products in their ration, and only one fed all their horses with oilseed by-products.

Commercial concentrates (based on mixtures of different energetic ingredients such as oats, corn, soybean meal, molasses, wheat middling, and vegetable oils) were used in the Chilean corralero horse diets, with 42.9% (21 of the 49) of the owners including 2.2 ± 2.0 kg daily. Mineral premix and vitamins were used in 181 horses from 32 of the studied farms, whereas 93 horses from 16 farms were not fed supplements. Finally, only 41 horses from 10 farms were fed different oils or fats, with linseed oil being the most popular.

One hundred and ninety-seven horses from 27 farms were dewormed between two and six times per year.

Of 275 horses, 149 horses (54.2%) did not suffer colic during the previous year, whereas 47 (17.1%) and nine (3.3%) horses suffered colic one or two times during the year before, respectively. There was a negative correlation (*r* = −0.72) between the numbers of colic episodes that the horses suffered per year and the amount of hay (other than alfalfa hay) offered, whereas the number of colic episodes that the horses suffered per year was positively correlated with corn grain (*r* = 0.6) and wheat middlings (*r* = 0.7).

Around 63% did not suffer laminitis disease the previous year, and 12% suffered laminitis during that period. The remaining interviewees did not know the answer. A negative correlation (*r* = −0.72) between the incidence of laminitis and the amount of hay was observed.

## 4. Discussion

Unfortunately, there is little scientific literature describing the characteristics of the Chilean corralero horse. In 1950, Denhardt [13] described the horse as a muscular, strongly built animal, with a broad chest and a good distance between its shoulders. Years later, a review of morphological characteristics of this breed during the rodeo sport season was reported by García et al. [14]. The girth of the Chilean corralero horse usually ranges between 162 and 182 cm for males and 164 and 184 cm for females [15]. In this study, the girth data fell within these ranges. Mares had a larger girth (168.3 ± 3.7 cm) than both stallions (165.5 ± 4.4 cm) and geldings (166.4 ± 4.3 cm), which was similar to the values obtained by García et al. [14], who reported 170.3 ± 7.1 cm for females and 168.8 ± 5.3 and 169.6 ± 6.4 for stallions and geldings, respectively. However, there is no established reference parameter for the scapula–ischial length (SIL) of this breed. In this study, the average SIL for mares was 153.1 ± 5.7 cm, while it was 150.6 ± 5.1 cm for geldings and 151.7 ± 4.9 cm for stallions. In the case of García et al. [14], mares had an SIL of 147.9 ± 6.3 cm, geldings had an SIL of 148.2 ± 7.9 cm, and stallions were shorter at a length of 145.6 ± 6.4 cm.

In this study, the wither height was not measured but information obtained from the study of Muñoz et al. [5] showed that the standard height of the withers of the breed established at 138–148 cm [15]. The morphological characteristics mentioned above indicate that Chilean corralero horse is considered a small horse. To estimate the average liveweight, the formula proposed by Carroll and Huntington [11] was used, and the estimated weight of the surveyed horses was 383.66 + 26.8 kg. Mares had the greatest estimated average weight of 394.16 ± 23.8, followed by geldings and stallions, with average weights of 379.34 ± 27.9 and 377.8 ± 25.8, respectively. The standard of the Chilean corralero horse establishes that mares are 2 cm longer in SIL than males due to their reproductive anatomy [16]. Several other studies about morphometric measurements of the Chilean horse are available for further information [5].

Stabling is a common practice in sport horse management and is part of the evolution of the horse throughout history, as it changed from being utilized primarily for agricultural and military purposes to use in sports and leisure [17]. This trend to confine the animal allows for a better control of the daily routine, feed intake, reproduction, and health management, among other benefits. To our best knowledge, there is limited information about Chilean corralero horse breeding and management, even during the reproductive or training phases. This research was conducted focusing on the sport phase of those horses exclusively focused on rodeo training. In the rodeo, as in any other equine sport discipline, horses are kept within single stalls which limit locomotion, making horse care easier and more economical [18]. However, the restricted motion and the lack of freedom to express characteristic behaviors may lead to stress and vices [19]. Upon the results of this study, it is clear from the routine handling management questions of Chilean corralero horses that 100% of the horses spent some time of the day within the stall (Table 2), usually more than 12 h a day. Another large percentage (59.3%) of the horses spent between a few hours to up to half of their day tied. Only a small percentage of the horses were left free in pens (8.7%) or paddocks (0.7%). These extensive confinement periods correspond to abnormal behavior and vices in the Chilean corralero horse and, according to Muñoz et al. [20], 10% of the horses studied exhibited these behaviors. Prolonged stabling can result in several undesirable behaviors such as “cribbing” (biting hard, often wooden surfaces, frequently swallowing air in the act) which can generate small fractures (less than 5 mm) in the horses’ teeth [21].

Daily exercise routines of the Chilean corralero horse were quite variable among farms. It seems that every trainer and/or owner managed their horses without following a common pattern within the discipline. Overall, Chilean corralero horses exercised six days per week, and the daily exercise consisted of walking, trotting, cantering, and bumping. Total daily exercise lasted 49.6 ± 27.8 min. The NRC [22] categorizes exercise as light, moderate, heavy, and very heavy for horses, depending on the mean heart rate, description, and types of event. In this case, the Chilean corralero horse training can best be categorized as heavy exercise, since, on average, the horse worked 4–5 h/week, during which time the activity was composed of 30% walk, 50–65% trot or canter, and 5% canter, jumping, or other skill. In this case, since there are no data on mean heart rate, bumping may be considered similar to jumping or cantering [22].

Horses are non-ruminant herbivores with an enormous capacity to digest and obtain energy from fibrous ingredients [23]. However, they have a small stomach (8% of the total gastrointestinal tract volume) and, thus, must eat small quantities many times throughout the day. In free-ranging conditions, horses spend between 16 and 20 h per day grazing and, thus, in nature, can consume small amounts of feed throughout the day [24]. Nevertheless, for practical reasons, owners of confined horses may not be able to simulate this feeding delivery [25]. Feeding frequency affects horse health and, thus, performance. In this study, a major portion of the animals surveyed (81.1%) were fed twice a day, whilst the remaining 18.9% were fed three times per day, which is within the recommendations for feeding horses to avoid colic [26]. Furthermore, the number of meals provided for the horses in this study was the same as the number of meals reported in a survey study of show-jumping horses [7], horses in the United Kingdom (UK) with high energy requirements [17], but differed from eventing horses [9] and racehorses [27], which received between one and five meals per day.

With regard to the type and amount of feed offered, this study found a wide variation in the diets and quantities of food offered, which suggests that the feeding management of these individuals is not formulated according to their requirements but rather according to the managers’ personal criteria. All horses surveyed had access to some kind of forage in concordance with recommendations by the NRC [22]. An average of 9.0 kg/horse (2.3% live bodyweight, “bwt”) of either alfalfa hay and/or pasture hay was offered daily; however, the exact amount varied greatly, between 5.6 and 19.1 kg/horse (as fed). More recently published recommendations for horses with high energy requirements indicate that hay intake should be 20 g dry matter (DM)/kg bwt/day [25]. Considering a 400 kg bwt for the Chilean corralero horse, the minimum forage intake should be 8 kg of DM daily. In contrast to high fiber feeding, 59% of the sampled horses received grains (oats or corn) and/or a commercial concentrate (locally produced or imported). These energetic ingredients were offered from 2.6, 1.3, 2.3, and 2.0 kg per day as fed for oats, corn, local, and imported commercial concentrate, respectively (Table 4). The average grain or concentrate offered was 2.1 kg/horse daily, which represents 0.5% of the horse liveweight. This value is in accordance with the recommendations of Owens [28] and the NRC [22]. Wheat by-products (wheat middlings and wheat bran) were also offered to horses as part of the daily diet as a replacement or partial replacement of energetic feedstuffs. The average offered was 2.1 kg/horse; nevertheless, the NRC [22] lists both by-products as concentrates (3.2 and 3.4 Mcal DE/kg, respectively) even although both are considerably higher in fiber than corn (ADF 15.5 and 12.1% respectively, compared to 3.4% ADF in corn) but similar to oat ADF content (13.5%). As the partial or total replacement of grain with wheat by-products did not follow a common pattern among farms, it is suggested that every owner follows their own criteria when establishing feeding practices for the Chilean corralero horse. Finally, when compared to the 400 kg bwt horses from the NRC tables [22], Chilean corralero horses are fed 2.8% of their bwt, which is slightly greater than the recommendation for horses in heavy exercise. It is worth noting that NRC recommendations do not account for breed-type behavior. Chilean corralero horses have a nervous and alert temperament which may increase the average energy requirements compared with a regular 400-kg horse [29]. Rosselot et al. [29] found that Chilean corralero horses tend to demonstrate proactive avoidance behaviors when presented with a challenge in a handling test; thus, this increased activity in response to environmental stressors could also impact nutrient requirements. Another factor that must be considered is the total rider and saddle weight that the horse supports on its back during competition and daily training. Muñoz et al. [5] conducted a study to determine the weight supported by Chilean corralero horses during a rodeo competition. The estimated back load maximum capacity of these Chilean corralero horses was 115.0 ± 6.1 kg, and the riders weighed on average 80.8 kg with a saddle weight of 11.6 kg; therefore, while the horse is not overloaded, the weight of the rider and saddle corresponds to 25% of the horse’s weight and, thus, has a lesser impact on its relative energy expenditure (25.7 Mcal/d DE) as compared to other sports with a lower rider/horse weight ratio such as show jumping and endurance, where the sum of weights for riders and saddle represents approximately 15% of the horses’ weight and the energy requirements.

In terms of nutrition, the use of fats and oils was not common, with just 17.8% of horses fed with some oil source. The amount varied greatly but averaged 30–60 mL/day. In general, corn oil was preferred for horse feeding since it was proven that, among vegetable and animal oils, it is the most used and accepted [30]. In contrast, mineral supplementation and the use of other additives/supplements are common practice within the industry. Sixty-five percent of horses were supplemented with mineral blocks, and 75.5% were given some supplement (liver protectors, vitamins premix, creatine sources, and joint supplements, among others).

In this study, colic events were negatively correlated to hay consumption and positively correlated to grain and wheat middling intake. Colic is a complex multifactorial condition and it has some association with feeds and feeding [31]. Grain overloading and, thus, high-starch diets along with low-fiber diets may predispose horses to colic [32]. In this study, grains were included in the diet with a high variation: 2.6 ± 1.6 kg/day of oats on average, ranging from 0.6–6.4 kg/day and 1.3 ± 1.3 kg/day of corn ranging from 0.2 to 3.6 kg/day. As mentioned before, the recommended amount of grain offered to a horse daily is 500 g/100 kg of liveweight. Thus, the Chilean corralero horse (400 kg liveweight) is expected to consume 2.0 kg of grains to supply the energy requirements (20–25 Mcal/d DE) according to NRC guidelines [22]. This recommendation comes from the limited ability of the enzymes in the horses’ small intestine to digest starch from cereal grains. When grain, which is high in starch, is overfed, part of the carbohydrates mentioned above are incompletely digested and escape absorption in the small intestine, traveling to the large intestine and promoting a disruption in the microbial population, leading to abnormal pH conditions which compromise intestine health [32]. These alterations may increase the risk of colic, laminitis, and other pathologies.

A characterization of the animal and its routine care was carried out through this study. This information complements all physics hypsometry studies that were done. To reach a better understanding of this breed’s nutritional requirements, more data should be taken in situ, such as time dedicated to working in each activity, as well as heart rate, blood metabolites, and other physiological variables.

Chilean legislation for animal welfare only establishes that owners should provide adequate feeding and healthcare to their animals, regardless the animal species. Therefore, the results of this study may provide information which could be considered when creating or enforcing legislation regarding horse care and training.

## 5. Conclusions

The average Chilean corralero horse spends approximately 12 h daily within a stall, and spends its remaining hours tied, loose in a pen, and/or training. The horse’s workload can be classified as moderate to heavy intensity according to the NRC [22], and includes six workouts weekly (walk/trot/canter) with two bumping workouts per week and two competitions per month.

During the training season, the diet of the Chilean corralero horse is based on forages, mainly alfalfa hay, plus oats as an additional energy source. Protein supplements such as oilseed by-products are used less frequently.

Additional studies are required to provide further information on nutritional requirements, feeding management, and exercise in order to improve the raising and management of this breed.

## Figures and Tables

**Table 1:** Characterization of horses included in the survey, concerning gender, age, and estimated weight calculated from the girth and scapula–ischial length (SIL).

Parameter						
Gender	*N*	%	Age (Years) (Mean ± SD)	Estimated Weight (kg) (Mean ± SD)	Girth (cm) (Mean ± SD)	SIL (cm) (Mean ± SD)
Geldings	84	30.6	10.9 ± 2.4 ^a^	379.3 ± 27.9 ^b^	166.4 ± 4.3 ^b^	150.6 ± 5.1 ^b^
Mares	90	32.7	9.7 ± 2.6 ^b^	394.2 ± 23.8 ^a^	168.3 ± 3.7 ^a^	153.1 ± 5.7 ^a^
Stallions	101	36.7	9.4 ± 2.6 ^b^	377.8 ± 25.8 ^b^	165.5 ± 4.4 ^b^	151.7 ± 4.9 ^ab^
All	275	100.0	9.9 ± 2.6	383.7 ± 26.8	166.7 ± 4.3	151.8 ± 5.3

^a,b^ Values within a column with different superscript letters differ significantly at *p* ≤ 0.05.

**Table 2:** Routine management of Chilean corralero horses.

Hours/Day	Number and Percentage of Horses			
	Stalls	Tied	Pens	Paddock
0–5	0	17 (6.2%) ^‡^	0	0
6–10	0	2 (0.7%) ^†^	0	0
11–15	172 (62.5%)	144 (52.4%) ^†^	24 (8.7%) ^†^	2 (0.7%) ^†^
15–19	17 (6.2%)	0	0	0
20–24	86 (31.3%)	0	0	0
Total	275 (100.0%)	163 (59.3%)	24 (8.7%)	2 (0.7%)

^†^ Animals that spent less than 15 h in a stall; ^‡^ animals that spent within 15 to 19 h in a stall.

**Table 3:** Exercise routine (minutes/workout) of Chilean corralero horses.

	Trot	Canter	Lunge	Bumping ^†^	Tournaments/Season
N	275	275	275	275	
Mean	8.6	22.3	2.5	16.3	
SD	6.6	6.1	8.7	6.4	
Time spent per activity (minutes/workout)					
0–15	78.5	6.9	93.5	37.8	
15–30	20.4	61.8	2.5	54.2	
30–45	1.1	31.3	3.3	8.0	
>45	0	0.0	0.7	0.0	
Activity (days per week): trot/canter/lunge					
Mean	5.6			2.4	8.6
SD	0.6			0.7	4.7

^†^*Bumping* refers to the exercise in which the horse performs lateral canter half-passes while maintaining contact at a 45° angle between the horse’s chest and the steer’s body.

**Table 4:** Feedstuffs by type and quantity that were offered to every horse on a daily basis, as fed.

	Horses N (%)	Farms N	Daily feed allowance	SD	Max	Min
**Forages (kg/d)**						
Alfalfa hay	122 (44.4)	22	8.8	2.3	19.1	4.9
Grass hay	67 (24.3)	13	9.2	1.7	14.3	5.6
Alfalfa cubes and/or pellets	64 (23.3)	8	8.6	0.9	11.3	7.1
Mix	22 (8.0)	6	10.7	2.6	15.7	7.0
	275	49				
**Cereal grains (kg/d)**						
Oats	57 (77.0)	14	2.6	1.6	6.4	0.6
Corn	17 (33.0)	4	1.3	1.3	3.6	0.2
	74	18				
**Wheat by-products (kg/d)**						
Wheat bran	93 (60.4)	19	1.9	1.2	5.3	0.5
Wheat middlings	61 (39.6)	11	2.4	1.3	6.0	0.7
	154	30				
**Oilseed by-products (kg/d)**						
Soybean meal	54 (90.0)	10	0.8	0.4	0.9	0.1
Canola meal	6 (10.0)	1	0.9	0.0	0.9	0.9
	60	11				
**Comercial concentrate (kg/d)**						
Locally produced	75 (83.3)	17	2.3	2.1	9.0	0.6
Imported	15 (16.6)	4	2.0	1.3	3.8	0.5
	90	21				
**Oils (ml/d)**						
None	226	39	0			
Corn oil	2	1	200 ^†^			
Sunflower oil	9	1	45			
Olive oil	2	1	50			
Linseed oil	26	4	60			
Soybean oil	5	1	40			
Fish oil	5	1	30			
**Minerals blocks**						
No addition	94	17 (34.7%)				
Addition	181	32 (65.3%)				
**Suplements (any kind)**						
No addition	53	12 (24.5%)				
**Addition**	222	37 (75.5%)				

^†^ Only at one farm during a limited period.
